# West Syndrome and the Importance of Routine Physical Examinations and Parental Education: A Case Report

**DOI:** 10.7759/cureus.31848

**Published:** 2022-11-24

**Authors:** Filipa Dias Freitas, Sofia Cavaco Raposo, Augusto Luis Nogueira

**Affiliations:** 1 Department of Family Medicine, Unidade de Cuidados de Saúde Personalizados (UCSP), Azambuja, PRT; 2 Department of Family Medicine, Unidade de Saúde Familiar (USF) Reynaldo dos Santos, Póvoa de Santa Iria, PRT

**Keywords:** hypsarrhythmia, epileptic encephalopathy, spasms, infantile spasms, west syndrome

## Abstract

West syndrome (WS), also known as infantile spasms, is a severe form of epileptic disorder of infancy and early childhood. It was first described by William West in 1841. Children with WS exhibit a triad of myoclonic-tonic seizures (spasms), a distinct electroencephalogram (EEG) pattern known as hypsarrhythmia and psychomotor development arrest. WS is classified into three main categories as symptomatic, idiopathic and cryptogenic based on etiological factors. The long-term prognosis depends on the etiological cause, but generally has a poor prognosis, and is associated with impaired development, neurologic structural anomalies, autism spectrum disorder and death. Treatment guidelines from the American Academy of Neurology and Child Neurology Society recommend that adrenocorticotropic hormone (ACTH) and vigabatrin are possibly effective in the cessation of spasms and hypsarrhythmia. We report an incidental diagnosis of WS in a six-month-old male baby that went to the Pediatric Emergency Department due to upper respiratory tract symptoms. The diagnosis was made after the development of spasms during a medical examination. This case highlights the importance of early diagnosis, parental education and prompt effective treatment as it may improve prognosis.

## Introduction

West syndrome (WA), also named infantile spasms, is a rare and severe epileptic encephalopathy, that usually starts around three to seven months of age, with a peak at six months [1,2]. It manifests by a triad of spasms, arrest of psychomotor development and hypsarrhythmia (an EEG pattern) [1,3]. It has an incidence of 2-3.5 per 10,000 live births and corresponds to 2-10% of all epilepsies in childhood [2-4]. It affects both genders, but a higher prevalence has been demonstrated in males (60:40) [3,4].

The spasms appear in clusters, most commonly on arousal from sleep, and involve muscles of the neck, trunk and limbs [1,4]. These spasms occur in two phases, the first one is characterized by sudden, brief contractions of one or more groups of muscles that take less than two seconds. It is followed by a tonic phase with a longer duration (two to ten seconds) [1,5]. They can occur in flexion, extension or flexion-extension and may be preceded or followed by episodes of crying and screaming. In some cases, eyes may be fixed or deviated during the crisis [1]. Other defining features are psychomotor development arrest and an interictal encephalogram (EEG) pattern known as hypsarrhythmia (first described by Gibbs and Gibbs in 1952) [6].

West syndrome is classified into three groups: symptomatic, when there is an identified etiology and/or a development arrest is present from the beginning of spasms; cryptogenic, when no known cause is present and the child has a normal development at presentation (this group usually has a better prognosis); idiopathic, when there is no identifying underlying cause (probably of genetic origin), no neurologic signs and symptoms [2,4,7]. The etiology of this syndrome can have prenatal, perinatal or postnatal origins. Prenatal causes comprise central nervous system malformations, neurocutaneous syndromes, intrauterine infections, metabolic disorders and chromosomal abnormalities. The perinatal causes include hypoxic-ischemic encephalopathy, neonatal hypoglycemia and perinatal strokes. Among the postnatal causes are trauma and infection [1,2,4]. The long-term prognosis is related to the etiological cause.

Treatment guidelines from the American Academy of Neurology and Child Neurology Society recommend that adrenocorticotropic hormone (ACTH) and vigabatrin are possibly effective in the cessation of spasms and hypsarrhythmia, although other antiepileptic medication and glucocorticoids are used and appear to be helpful [8].

## Case presentation

A six-month-old male baby presented to the Pediatric Emergency Department (PED) with the following clinical condition: a fever peak (rectal temperature of 38.7ºC), malaise, irritability and nasal congestion for a day.

Regarding the patient’s personal history, we highlight the fact that he had a heterozygous twin brother (dichorionic diamniotic pregnancy complicated by cholestasis), they were the firstborns of both parents, born at 36 weeks and five days of gestation, after a dystocic delivery (by forceps), with normal values for weight and Apgar score. During the neonatal period, the only incident was the development of jaundice that required phototherapy.

Until the moment he presented to the PED, he grew in the 3rd percentile of weight, length and cephalic perimeter and he used to visit both his family doctor and pediatrician. After consulting the clinical notes of the medical appointments, we noticed that he had been diagnosed with plagiocephaly at three months of age, torticollis at four months of age and showed axial hypotonia at five months of age. He had started attending nursery school at the beginning of that month, took no regular medication and his national vaccination programme was up to date.

Regarding family history, both parents were healthy, non-blood-related and the only noteworthy information was a history of controlled epilepsy of the maternal grandmother. There was no record of any other neurologic or hereditary diseases in the family.

On examination in the PED, it was noticed that the baby had an asymmetric brachycephaly, and was not able to direct his eyesight, stare or follow an object with his eyes. He had a preferential look directed to the right. He assumed a bizarre posture, with elbow flexion and hyperextension of inferior limbs. Axial hypotonia was also present, with no cephalic control. There was also distal hypertonia in which his hands were mostly closed. During examination clusters of flexion-extension symmetrical spasms occurred more than once.

After better exploration of this apparent neurologic condition, parents described that for one month they had been noticing episodes of flexion of the trunk and limbs, in clusters of seven to eight seconds, about three to four times per day, while awake, most frequently in the last week and associated with irritability. They thought they were startling reactions and told the doctor that the twin brother presented with the same condition.

**Figure 1:**
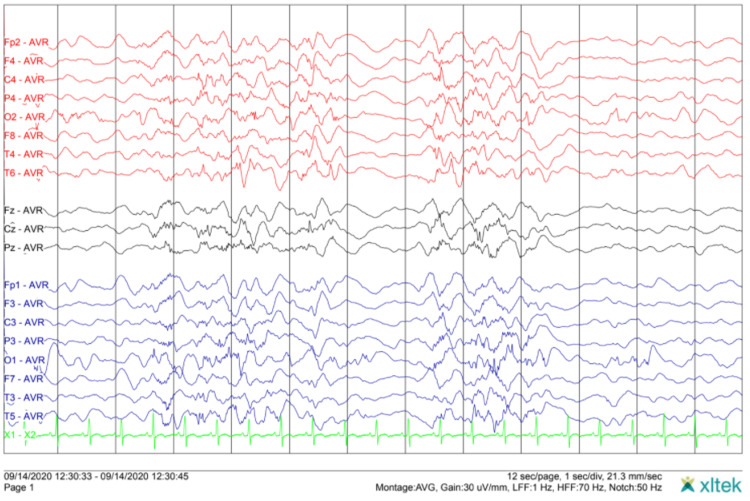
EEG performed on Pediatric Neurology Department showing a pattern known as hypsarrhythmia

Laboratory analysis (Table 1) and cranioencephalic computed tomography in the PED were normal and the twins were transferred to a tertiary pediatric hospital, with a neuropediatric department, to make an electroencephalogram (EEG). EEG (Figure 1) showed a pattern of hypsarrhythmia with multifocal paroxysmal activity and multiple spasms, in clusters, compatible with West syndrome, which was also confirmed for the twin brother.

**Table 1:** Laboratory results from the PED PED: Pediatric Emergency Department

Analysis	Values
Hemoglobin	12.1 g/dL
Leukocytes	13300/µL (Neutrophils: 6210/µL- 46.7%; Lymphocytes: 6410/µL- 48.2%)
Platelets	471000/L
Creatinine	0.42 mg/dL
Urea	19 mg/dL
Ionogram	Sodium: 142 mmol/L, Potassium 4.45 mmol/L
Lactic dehydrogenase	399 UI/L
C-reactive protein	<0.5 mg/dL
Phosphate	6.1 mg/dL
Total Calcium	9.9 mg/dL
Uric acid	4 mg/dL
Aspartate transaminase	51 UI/L
Alanine transaminase	44 UI/L

## Discussion

West syndrome (WS) was first described in 1841 [2,3]. Even though this was over 180 years ago, it is still challenging to diagnose and no known treatment exists. It is a rare and severe epileptic encephalopathy that starts in early childhood [1,2]. It presents with a triad of spasms, arrest of psychomotor development and the EEG pattern known as hypsarrhythmia [1,3]. The spasms, at this stage of life, are not easy to detect for either parents or even health care professionals, which may delay the beginning of the investigation and, consequently, the diagnosis. Therefore, education of families and physicians is important to shorten the delay from the start of symptoms to the diagnosis and the beginning of treatment, which probably leads to a better prognosis [1,2]. The spasms may go unnoticed and be mistaken for colic or gastroesophageal reflux episodes. In most cases, the beginning of the investigation depends mostly on parental evaluation and videos of these events help on it [2]. The prognosis of patients with WS is still poor and is related to the etiology, age of presentation and late or inadequate treatment [1].

WS is classified into three main categories as symptomatic, cryptogenic and idiopathic based on their characteristics and etiological factors. The etiology of this syndrome can have prenatal, perinatal or postnatal causes [2,4,7]. In our case report, the patient seems to belong to the symptomatic group because he presents with a development arrest since the beginning of the presentation despite an unknown etiology. From his personal history, no cause is suspected, further investigation is needed in order to find one.

The diverse etiological factors seem to generate a stress response with increased production of corticotrophin-releasing hormone from the immature brain, which results in epileptic spasms. This action can be inhibited by adrenocorticotropic hormone (ACTH), which represents one of the most effective medications for the cessations of spasms and hypsarrhythmia (along with vigabatrin, other anticonvulsants and glucocorticoids) [4]. Short-term treatment with ACTH has shown promising results in a trial of infants with WS [3,9]. Vigabatrin seems to be more effective for infantile spasms related to tuberous sclerosis [10].

From this case we emphasize the need of paying attention to parents’ worries, actively asking for changes in children’s behavior and doing a complete clinical history and physical examination.

## Conclusions

West syndrome is a rare epileptic disorder with a significant impact on psychomotor development. Early detection and referral to a pediatric neurologist is key for clinical diagnosis and effective treatment, since this may improve the prognosis. Subsequent follow-up should be maintained.

It is important to be alert to some of the signs and symptoms when talking to parents and during physical examination. Parent education and awareness is critical for early diagnosis. Although some of the medications are helpful (especially if initiated early on), the prognosis is still poor.
